# The growing impact of human papilloma virus (HPV)-associated cancers in men in Costa Rica: epidemiological and economic burden

**DOI:** 10.3389/fpubh.2025.1487256

**Published:** 2025-06-18

**Authors:** Sebastian Ospina-Henao, Francisco Brenes-Castillo, Marcel Marcano-Lozada, Maria Alejandra Betancur-Díaz, Sebastian Medina, Helena Brenes-Chacon, Maria L. Avila-Aguero

**Affiliations:** ^1^Faculty of Medicine, Universidad de Ciencias Médicas (UCIMED), San José, Costa Rica; ^2^Psychological Research Institute (IIP), Universidad de Costa Rica (UCR), San José, Costa Rica; ^3^Medical Affairs Department, Merck Sharp and Dhome (MSD), Central America and the Caribbean, San José, Costa Rica; ^4^GMVC, Merck Sharp and Dhome (MSD), Bogotá, Colombia; ^5^Pediatric Infectious Diseases Division, Hospital Nacional de Niños “Dr. Carlos Sáenz Herrera”, Centro de Ciencias Médicas, Caja Costarricense de Seguro Social (CCSS), San José, Costa Rica; ^6^Center for Infectious Disease Modeling and Analysis (CIDMA), Yale University New Haven, New Haven, CT, United States; ^7^Instituto de Investigación en Ciencias Médicas (IICIMED), San José, Costa Rica

**Keywords:** human papilloma virus, human papilloma virus related disease, male HPV-disease, HPV-related cancer, anal cancer

## Abstract

Human papillomavirus (HPV) is the most common sexually transmitted infection in men and women and is responsible for a substantial burden of disease worldwide. Although HPV-related disease burden is high in women due to cervical related disease and cancer, men are directly affected by HPV. According to the World Health Organization (WHO), one in three men has a prevalent HPV infection worldwide. Currently, there is a lack of data regarding the epidemiology and healthcare resource utilization (HCRU) of HPV-associated cancers among Costa Rican men. This study aimed to describe the epidemiological characteristics of HPV-associated male cancer and disease and the HCRU in Costa Rica. HPV-related cancers in men were assessed through retrospective database study for epidemiology and Delphi panel with five experts for HCRU. A total of 1,340 men with penile, anal, and head and neck cancers between 2012 and 2016 were identified in the database, with a mean age of 63.6 years. Anal cancer accounted for 48% of cases, followed by head and neck 44%, and penile cancer 11%. The cumulative rate of HPV-associated cancer in men per 100,000 population increased from 8.6 in 2012 to 55.5 in 2016. According to 4/5 panelists, resources for the disease management were also scarce. Panelists agreed that the cost for HPV management within their institution was 0.6–40,000 USD. Despite the increasing incidence e of HPV-related cancer in men, HPV prevention in men continues to be an under-served issue in public policy that could result in substantial economic and clinical burden. National health authorities should promote strategies to prevent HPV infections and associated diseases among Costa Rican men.

## 1 Introduction

Human papillomavirus (HPV) is a double-stranded circular DNA virus with more than 400 genotypes identified worldwide. HPV has a specific tropism for epithelial cells and causes a wide variety of diseases that can range from clinically asymptomatic infections to proliferative lesions that can progress to neoplasia and cancer (1, 2). The main source of HPV transmission is sexual activity, but not the only one (3). In recent years, a surge in HPV-related cancer and diseases has been examined in high-income countries, especially in oropharyngeal, penile, and anal cancers (4).

The global prevalence of HPV DNA in neoplastic samples has increased due to vigilance, among other things, from 20.9% in 1990 to 65.4% in 2000 (5). Approximately 45,000 HPV-associated cancers are diagnosed annually in the United States, with 60% detected in women and 40% in men. The overall incidence of cancer associated with HPV infection in men in 2017 was 11 per 100,000 (6).

Studies have found that HPV-related cancer and diseases have an economic burden on healthcare systems (7–9). In 2010 in the United States, the oropharyngeal cancer, anal cancer, penile cancer, and genital warts associated to HPVHPV represented a annual cost of $306, $155, $7, and $288 millions of U.S. dollars, respectively (7). Between 2002 and 2015 in the Republic of Korea, the healthcare cost of HPV-related cancer and diseases in male patients went from $6.9 million to $47.1 million (8).

In Costa Rica, HPV-related cancer and diseases have a high incidence among Costa Rican women, but information on the clinical and economic burden and healthcare resource utilization (HCRU) among Costa Rican men are not well known (9). Acquiring more HCRU information and data on economic burden among HPV-associated cancer and diseases may aid decision-makers to better allocate health care resources and estimate the impact of public health interventions, such as by developing more effective gender-neutral vaccination schemes (10). In Costa Rica, HPV vaccination coverage to the general population started after this study was performed; HPV vaccination began in 2019 in 10-year-old girls, with no catch-up strategy. Vaccination coverage in 2019 was 98–95% for the first and second doses, respectively; however, it decreased during the pandemic years, reaching an increase of 74–69% in 2022, respectively. Gender-neutral vaccination begins in 2023, with coverage in men of 40%.

Genotype 16 predominates in women with high-grade lesions and cancer in ~45% of the samples analyzed, genotype 18 occupies second place in women with cancer, while in men, no information is still available.

This study aims to describe the epidemiological characteristics of HPV-associated male-cancer, and to describe the HCRU and total direct medical cost per person after diagnosis of HPV-associated male cancer by age year and the associated cumulative medical costs using a Delphi panel analysis.

## 2 Materials and methods

### 2.1 Study design

National information on HPV-associated male-cancer and disease in patients older than 18 years-old was retrospectively gathered between January 2012 and December 2016 from the Costa Rican Social Security System CCSS). The CCSS provides healthcare to ~96–97% of the population. As a mandatory reportable disease, available information of HPV infection in this database is representative of the Costa Rican population. The database was used to analyze epidemiological information of patients, incidence of disease, anatomical location (anus, oropharynx, larynx, oral cavity, and penis). ICD-10 codes for HPV-related cancer and diseases in men were used as showed in Supplementary Table S1.

Due to the novelty of this topic, a Delphi method analysis (a well-established research tool used for consensus building) was performed (12), with the participation of five experts to evaluate healthcare resource utilization. Specialists had at least 3 years of experience in the fields of oncology, urology, and otorhinolaryngology.

The Data collection instrument was adapted from the instrument designed and developed by Berrada et al. (11) and translated into Spanish. A specialist in our team assessed the wording of each question, definition of the response variables, nature of values, measured ranges, and scales used (Appendix I).

The Delphi panel used consecutive rounds of surveys to reach consensus on specific questions. Advantages of this method lay in the anonymity, controlled feedback, iterative process, and statistical synthesis of quantitative information. Since anonymity is maintained throughout the process (during each round of comments and arguments), the knowledge of the person making each comment does not produce a bias in the opinion of the rest of the participants (12). Furthermore, after each round, another independent evaluator offered a synthesis of the experts, comments, as well as their arguments. As the rounds progress, consensus is reached, and the process stopped once consensus has been achieved among most participants and the average of the ratings expressed determines the outcome (≥70% consensus).

Participants were contacted via email. The first questionnaire was sent as a link via email and asked for participant's sociodemographic characteristics and work-related information. Clinical information about HPV patients, institutional instruments to guide decisions, and management of patients and outcomes were also included in the survey (see Appendix 1 and 2 with both questionnaires). Questions were continuous (i.e., when a number was required as a response) or categorical (Yes vs. No, or Never vs. Sometimes vs. Regularly vs. Always). The second round of questionnaires was also sent as a link via email and contained average responses from round 1 questions that reached no consensus (under 70%) for categorical variables, questions where an agreement was required (for continuous variables), or questions that needed further clarification. Participants could either assess whether they agreed with the average responses (Yes vs. No) or whether they wanted to modify their opinion. Panelists were invited to briefly justify their new responses. The third round followed the same procedure as the second round, but the questions were written on the email.

Part of this study was conducted during the COVID-19 pandemic, nevertheless, no contingency measures were implemented to manage study conduct because of the pandemic.

Primary endpoints collected from Delphi panel were the identification of an average healthcare resource utilization (HCRU) per year in patients with HPV-associated male-cancer and disease diagnosed in Costa Rica, including costs of disease, hospitalization, treatment, and burden of disease. Secondary outcomes from national database were annual standardized case incidence and hospitalization rate.

### 2.2 Data analysis

#### 2.2.1 Incidence and cumulative disease rate

For HCRU Data from the Delphi Panel, first questionnaire was analyzed after all five experts completed their responses using Microsoft Office Excel (version 16.77.1, Redmond, Washington, USA). Categorical variables were analyzed using frequencies, and continuous variables were summarized through mean (standard deviation) or median (interquartile range 25–75) according to normality. For the Delphi panel analysis, agreement was reached when a category reached 70% or more. Reporting average responses through medians and ranges is a common approach in Delphi Panels, since this provides an estimate of group tendency (13). Missing values were reported, and missing data was not imputed.

HCRU and costs were estimated based on the average responses captured from the Delphi panel. Incident method was employed to assess direct costs associated with HPV-associated male-cancer and disease. Unit costs were obtained using two approaches: a “local” approach using local cost of each healthcare resource; and a “global” approach (in the absence of local unit costs) where costs from comparable countries with available costing data were assigned to healthcare resources and converted to local currency. Wherever possible, unit costs were identified locally either from hospital list or health insurance data. Cost was expressed in local currency but converted to purchasing power parity (PPP) U.S. dollars using the World Bank conversion rate at the time of analysis (14).

### 2.3 Ethics review

This study complies with the Declaration of Helsinki, the current Costa Rican legislation, and had approval of the institutional review board and the scientific ethical committee (Protocol Approval Number: CEC–UCIMED 647-07-2023). Given the source of data, the need to obtain informed patient consent was waived.

## 3 Results

### 3.1 Patient characteristics

**Figure 1:**
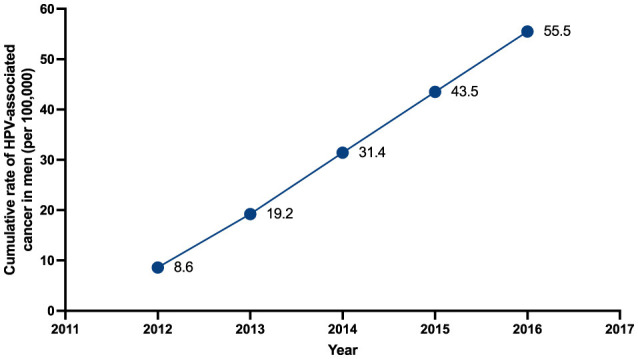
Cumulative rate of HPV-associated male-cancer from 2012 to 2016.

From 2012 to 2016 a total of 1,340 patients with a new diagnosis of HPV-associated male-cancer and diseases received consultation at the CCSS. Mean age was 63.6 (SD: 14.15) years of age. Based on the estimated male population of Costa Rica (according with data form the Central American Population center), the cumulative rate of HPV-associated male-cancer and diseases increased during the study period, with rates starting at 8.6 cases per 100,000 males in 2012, to a cumulative rate of 55.5 cases per 100,000 males in 2016 (Figure 1).

Most diagnosis of HPV-related malignancies were documented in the anal 649/1,341 (48.4%) followed by larynx 273/1,341 (20.4%), oropharynx 144/1,341 (10.7%), penis 142/1,341 (10.6%), and oral cavity 133/1,341 (9.9%), with no differences among age groups or by year.

**Figure 2:**
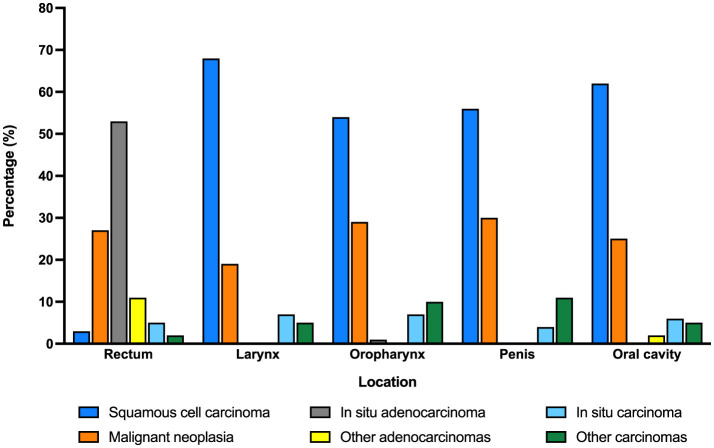
Distribution of HPV-related male cancer by histologic diagnosis and location.

Histologic diagnosis also varied according to the anatomic location of the malignancy (Figure 2). While squamous cell carcinoma was the most frequent type of cancer in most locations (larynx, oral cavity, penis, and oropharynx), adenocarcinoma was the predominant malignancy in anal HPV-associated disease.

### 3.2 Delphi panel findings

A Delphi panel evaluation was used to examine the HCRU and direct medical costs after diagnosis of HPV-associated male-cancer. Delphi panel participant characteristics are described in Table 1.

**Table 1:** Delphi panelist characteristics.

**Physician and practice characteristics (*****n*** = **5)**	
**Physician characteristics**	
Age, median (range) years	45 (34–49)
Sex, male (%)	4 (80%)
Specialty, *n* (%)	
Oncology	3 (60%)
Urology	1 (20%)
Otorhinolaryngology	1 (20%)
Years in specialty practice, median (IQR)	13 (4–20)
**Practice characteristics**	
Patients seen per month, median (IQR)	200 (75–210)
Duration of care for HPV related cancers and diseases in years, median (IQR)	15 (4–20)
Male AGW patients, median (IQR) percentage	70 (50–100)
First presentation of HPV cancers and related diseases	80 (20–100)
Prevalence of HPV diagnoses within participants' institution, median (IQR)	10 (4–11.2)

Regarding diagnosis, routine tests for HPV-associated male-cancers were never/sometimes applied within the practice of 4/5 (80%) panelists. The specialist in head and neck cancer regularly implemented routine tests. However, 1/5 (20%) mentioned that they regularly/always provided routine diagnostic tests for HPV, including biopsy, polymerase chain reaction (PCR) test for HPV, cytology, anoscopy, and penoscopy among other procedures. Biopsy was the most common diagnostic procedure test used. Among panelists, 4/5 (80%) did not routinely test sexual partners for HPV, even though the prevalence of the disease was described as 50 (IQ: 40–200) cases of HPV-associated male-cancer and disease per year.

All experts responded that patients with HPV-associated male-cancer and disease regularly/always receive specific treatment, such as cryotherapy (2.5, IQ: 0–4) and laser surgery (0.5, IQ: 0–2). Therapy is chosen by morphology of lesion and size. Chemotherapy was considered a second line treatment in 60% of responses (3/5) in the treatment of HPV-associated male-cancer and disease.

Need of multidisciplinary evaluation was mentioned by 4/5 physicians, mainly regarding otorhinolaryngology, surgery, and urology specialists.

In terms of hospitalization, 4/5 physicians described that they never/sometimes had an HPV patient hospitalized for more than 24 h. Among those hospitalized, median days of hospitalization was 7 (IQ: 0–7). Regarding complications, 4/5 (80%) panelists mentioned that patients regularly/always had side effects, such as a burning sensation in their lesions. Complications such as bleeding, infections, and anatomic sequelae among others, were never/sometimes reported.

Regarding follow-up, a median of 4 consultations (IQ: 3–50) were required by patients with HPV-associated male-cancer and disease per year, and recurrence of disease was described in 15 (IQ: 4–50) patients, and therapies such as radiotherapy, surgery, and prolonged follow-up was used by 4/5 (80%) experts.

Interestingly, 4/5 (80%) panelists agreed that there is a lack of written guidelines in the country to manage HPV-related cancer and diseases. 4/5 (80%) panelists described that they did not have sufficient resources to capture and treat patients with HPV-associated male-cancer and diseases. Some of the reasons mentioned were inadequate working conditions, lack of access to medical care, low availability of diagnostic tests, and lack of treatments.

Related to annual direct costs of HPV-associated male-cancer and diseases, panelists agreed that close to 0.6–40,000 USD were expended to treat patients within their institutions, 0.1–20,000 USD to treat patients at external consultations, 0–10,000 USD to treat HPV patients at internal consultations, and close to 0.6–30,000 USD in HPV-related treatment costs.

## 4 Discussion

The impact of HPV-associated cancers in men is increasingly significant, with a notable rise in the incidence of certain cancers. HPV is a well-established cause of various cancers, including oropharyngeal, anal, and penile cancers in men. However, the epidemiology and impact of the infection have not been sufficiently studied, and vaccination rates in this population have not been improved.

In this study we identified 1,340 male patients with diagnosis of HPV-related cancer between 2012 and 2016 in the Costa Rican Social Security System. During the study period, cumulative rate of disease increased significantly, from 8.6 in 2012 to 55.5 cases per 100,000 males in 2016. Anal and larynx were the most common anatomical places for lesions with squamous cell carcinoma as the most common histologic type of malignancy in most locations. These findings were similar to global information regarding burden of HPV-associated cancer and diseases (15–18). Age at diagnosis in our cohort was congruent with described characteristics of HPV-male related disease in literature (19). Costa Rica started HPV vaccination in 2023, so it is not yet possible to obtain impact data.

The increased incidence of HPV among men may be related to socio-behavioral factors, a historical lack of awareness, changing sexual practices, and disparities in vaccination rates. The main reasons are (20, 21):

- Lack of awareness and screening: historically, there has been less awareness of HPV in men than in women. HPV is often considered a women's health issue because of its link to cervical cancer, resulting in a lack of routine screening and education for men.
- Increased sexual activity: changes in sexual behavior, including an increase in the number of sexual partners and the prevalence of unprotected sex, have contributed to a higher rate of HPV transmission among men.
- High prevalence among MSM: men who have sex with men (MSM) are at increased risk of HPV infection. This increased risk may be attributed to biological factors, riskier sexual practices, and lower frequency of screening for HPV-related diseases.
- Vaccination rates: Although HPV vaccines are available and recommended for boys and young men, vaccination rates have historically been lower compared to girls and young women, contributing to higher incidence rates among unvaccinated populations.
- Increased testing and detection: due to increased awareness and testing, more cases of HPV are now identified in men than in previous years. This increase in detection may not necessarily indicate a higher incidence, but rather a better diagnosis.

Histologic findings in biopsy samples consistently found that all age groups had squamous cell carcinoma in penis, head, and neck. In turn, this correlates with the etiology of HPV, which produces these lesions (19, 22). Nevertheless, adenocarcinoma was the main histological type in more than 50% of anal cancers. Previous studies identified that HPV may infect the glandular mucosa of the colon and there is a possible association between HPV and coloanal cancer (23, 24).

Limitations for diagnosis, management, treatment, and follow-up of HPV-disease were described by the expert panelists. Screening programs are recommended practices to test and treat HPV associated diseases, especially within low and middle-income countries (25). The higher risk of HPV related diseases and genital cancer in men, must be considered for clinical practices (26). In developing countries, the high incidence of HPV-disease is mainly associated with the lack of resources devoted to screening programs and no access to HPV vaccines (27).

The economic burden of human papillomavirus (HPV)-related diseases in men, particularly with the increasing incidence of oropharyngeal and anal cancers, is substantial and multifaceted, encompassing both direct and indirect costs. Direct costs include healthcare expenses related to diagnosis, treatment, and management of these cancers. For instance, the healthcare costs for HPV-related cancers in Norway were significant, with oropharyngeal cancer incurring the highest total cost per patient over the first 3 years following diagnosis (28). Similarly, in France, the total economic burden associated with HPV-related cancers was substantial, with oropharyngeal cancer alone accounting for a significant portion of the costs (29).

Indirect costs primarily involve productivity losses due to morbidity and premature mortality. In Sweden, indirect costs constituted a large portion of the total economic burden of HPV-related cancers, with premature mortality being a major contributor (30). The societal cost of oropharyngeal cancer, particularly HPV-positive cases, is also significant, with indirect costs forming a substantial part of the total cost (31). A 2006 French study, identified that cost for outpatient treatment for head and neck cancer in men was around 110.5 million euros. In turn, the total costs of HPV-associated cancer in men were 107.2 million euros, mainly due to head and neck cancer (32).

Our study is the first to present the direct and indirect costs associated with HPV disease in men in Costa Rica and Central America, and as reported in the literature, it shows a high cost for social security.

It is important to mention that the economic burden varies significantly across different regions due to variations in healthcare systems, cancer incidence rates, and access to HPV vaccination and prevention programs (33, 34).

Our study had several limitations. Given the retrospective nature of the study, incomplete data could have been identified, nevertheless, most general characteristics of population were gathered correctly. Although an objective of the study, mortality of HPV-associated male-cancer and disease was not available for the analysis if this study. Since this study was performed using national data, available information was limited by operational aspects of the data collection. As there was only one clinical history record, it was not possible to differentiate whether patients were treated in outpatient or inpatient settings; we were also unable to determine how many of the patients with confirmed HPV infection had any of the associated cancers analyzed. The possibility to have this information from the Delphi panelists given their extensive experience in the topic helped overcome this limitation. Only five participants were part of the Delphi panel which could have led to bias. However, participants were selected for having expert knowledge in HPV among Costa Rican men.

According to the WHO, one in three men worldwide over the age of 15 are infected with at least one type of HPV. Most cases are asymptomatic, but they can lead to long-term sequelae and mortality. Burden of disease in women is better understand and efforts have been carried out to increase awareness and prevention. Nevertheless, perception of disease differs among men, even when the number of cases among men can be as high as 70,000 per year (35–38). This study emphasizes the need to have up-to-date information in Costa Rica on HPV-related cancer and diseases among men and the burden of this disease within Costa Rica.

The findings of this study identified the need for future directions in the fields of research, prevention, awareness, and integrative global health. Continued research on HPV's role in various malignancies, their risk factors, and effectiveness of treatment strategies is essential to improve outcomes and survival. Public education campaigns targeting men are needed to improve understanding of HPV disease. Expanding access to HPV vaccination programs for both men and women can significantly reduce the incidence of HPV-associated cancers and diseases in the future. Routine incorporation of preventive measurements and screening in medical practice are necessary for men, alongside with women.

## 5 Conclusion

The burden of cancer and HPV-associated diseases in men is a growing public health problem with significant epidemiological and economic consequences. In Costa Rica alone, 1,340 male patients were diagnosed with cancer and HPV-related diseases in a 4-year period, with an increasing cumulative rate of disease in this cohort. Recognizing the magnitude of the problem and implementing effective prevention are crucial to protecting men's health and reducing the future burden of these cancers. Despite the increasing burden of HPV-associated cancers in men, vaccination rates remain low. This low coverage underscores the need for increased efforts to promote HPV vaccination among males, which has been shown to be effective in preventing HPV-related diseases.

Overall, the growing impact of HPV-associated cancers in men highlights the importance of continued surveillance, increased vaccination efforts, and targeted prevention strategies to mitigate this public health challenge.

## Data Availability

The original contributions presented in the study are included in the article/Supplementary material, further inquiries can be directed to the corresponding author.
