# Supplementary material for: The growing impact of human papilloma virus (HPV)-associated cancers in men in Costa Rica: epidemiological and economic burden
Source: Front Public Health. 2025 Jun 18;13:1487256. doi: 10.3389/fpubh.2025.1487256 (PMC12213727; doi:10.3389/fpubh.2025.1487256)
Supplement: Supplementary file 1 [file Supplementary_file_1.docx]

**Table S1. ICD-10 diagnosis codes for HPV-related cancers in men**

| **HPV-related cancers** | **ICD-10 codes** |
| --- | --- |
| Penile cancer | C60.0, C60.1, C60.2, C60.8, C60.9 |
| Anal cancer | C21.0, C21.1, C21.2, C21.8 |
| Head and Neck: Oral cavity | C02.0, C02.1, C02.2, C02.3 C02.9, C03.0, C03.1,  C03.9, C04.0, C04.1, C04.8, C04.9, C05.0, C06.0,  C06.1, C06.2, C06.8, C06.9 |
| Head and Neck: Oropharynx | C01.9, C02.4, C02.8, C05.1, C05.2, C09.0, C09.1, C09.8, C09.9, C10.0, C10.9, C10.8, C10.9, C14.0, C14.2, C14.8 |
| Head and Neck: Larynx | C32.0, C32.1, C32.2, C32.3, C32.8, C32.9 |
