# Supplementary material for: The growing impact of human papilloma virus (HPV)-associated cancers in men in Costa Rica: epidemiological and economic burden
Source: Front Public Health. 2025 Jun 18;13:1487256. doi: 10.3389/fpubh.2025.1487256 (PMC12213727; doi:10.3389/fpubh.2025.1487256)
Supplement: Supplementary file 2 [file Data_Sheet_1.docx]

**Appendix**

Appendix I: Questionnaire for Round I

I 1 On average, how many patients do you provide care to per month?

2 On average, how many patients with diseases and cancers related to the Human Papillomavirus do you see per month?

3 Time providing care to patients with diseases and cancers related to the Human Papillomavirus: (years)

4 Of your patients with diseases and cancers related to the Human Papillomavirus, what percentage are: (a) men, (b) women?

5 Of your patients with diseases and cancers related to the Human Papillomavirus, what percentage is under 20 years old?

6 Of your patients with diseases and cancers related to the Human Papillomavirus, what percentage presents the condition for the first time?

7 Of your patients with diseases and cancers related to the Human Papillomavirus for the first time, what percentage are: (a) men, (b) women?

8 Of your patients with diseases and cancers related to the Human Papillomavirus for the first time, what percentage is under 20 years old?

9 How many cases of men diagnosed with diseases and cancers related to the Human Papillomavirus are there in Costa Rica annually?

10 In the United States, the prevalence of diseases and cancers related to the Human Papillomavirus in men is approximately 11.2 What do you consider to be the prevalence of diseases and cancers related to the Human Papillomavirus at your institution?

IIA 11 At your institution, what department(s) offers care to patients with diseases and cancers related to the Human Papillomavirus?

12 In addition to the clinical review, how often does your institution perform diagnostic tests on patients with diseases and cancers related to the Human Papillomavirus? (never, sometimes, regularly, always)

13 How often are the following diagnostic tests ordered for patients with suspected HPV-related diseases and cancers at your institution? HBV serology, HCV serology, ELISA for HIV, VDRL for syphilis, biopsy, PCR for HPV, cytology, cervical, penoscopy, anoscopy, others (never, sometimes, regularly, always)

14 In men with diseases and cancers related to the Human Papillomavirus, what are the diagnostic procedures used in your institution and the

average number of visits for each procedure? (please rank from most used to least) Biopsy, simple observation, other (specify) (range of, average of visits)

15 In your institution, is a systematic review of sexual partners of patients with diseases and cancers related to the Human Papillomavirus carried out? (yes, no)

16 In your institution, are laboratory tests systematically requested from the sexual partners of patients with diseases and cancers related to the Human Papillomavirus? (yes, no)

17 If yes, which of the following are the most requested? (choose the 3 most requested and rate from 1 (most requested) to 3 (least requested)) HBV serology, HCV serology, ELISA for HIV, VDRL for syphilis, biopsy, PCR for HPV, penoscopy, anoscopy, other.

18 At your institution, are there clinical practice guidelines available for the treatment of diseases and cancers related to the Human Papillomavirus (yes, no)

19 Do you have all the necessary resources for the treatment of diseases and cancers related to the Human Papillomavirus according to the guidelines? (yes, no)

20 If the answer is “no”, what resources are missing?

IIB 21 At your institution, how often do patients with diseases and cancers related to the Human Papillomavirus receive a specific type of treatment? (never sometimes, regularly, always)

22 At your institution, what type of therapy is considered the first line of treatment for diseases and cancers related to the Human Papillomavirus? Pharmacological treatments, ablative treatments.

IIC 23 Whether pharmacological or ablative, which treatment is most commonly prescribed as a first line of treatment at your institution? And what is the average number of visits for each one? (please rank from the most prescribed to the least)

Pharmacological: Trichloroacetic acid, Podophyllin, Imiquimod, Others.

Ablative: Cryotherapy, Excision, Laser Surgery, others. [Rating (1 for most commonly prescribed). Average number of visits]

24 For what reason(s) is this choice made at your institution? (choose top 3 reasons and rank from 1 (most important) to 3 (least important)) Number, size, site and morphology of lesions, convenience, patient preference, adverse effects, cost of treatment, provider experience.

25 At your institution, what type of therapy is considered the second line of treatment for diseases and cancers related to the Human Papillomavirus?

26 At your institution, how often are patients with diseases and cancers related to the Human Papillomavirus hospitalized for more than 24 hours for treatment? (never sometimes, regularly, always)

27 How many days on average do patients with diseases and cancers related to the Human Papillomavirus stay in the hospital? (days)

28 How often do patients treated for diseases and cancers related to the Human Papillomavirus have side effects? (never sometimes, regularly, always)

29 Do patients treated for diseases and cancers related to the Papillomavirus have the following side effects? Burning, itching or swelling, crusting, discoloration, other (never, sometimes, regularly, always)

30 Do patients treated for diseases and cancers related to the Human Papillomavirus have complications? (never sometimes, regularly, always)

IID 31 What is the total annual cost of a patient with a disease and cancer related to Human Papillomavirus at your institution? (answer in colones)

32 What is the annual cost of a patient with a disease and cancer related to the Human Papillomavirus who attends an outpatient clinic at your institution? (answer in colones)

33 What is the annual cost of a patient with a disease and cancer related to the Human Papillomavirus who is treated in an internal consultation at your institution? (answer in colones)

34 What is the annual cost of the treatments received by a patient with a disease and cancer related to the Human Papillomavirus at your institution? (answer in colones)

III (Results of the management of diseases and cancers related to the Human Papillomavirus (side effects, complications, referrals to other specialties, follow-up and recurrences). 35 How often do patients treated for diseases and cancers related to the Human Papillomavirus have the following complications? bleeding, infection, deformity, others (never, sometimes, regularly, always)

36 At your institution, how often are patients with diseases and cancers related to the Human Papillomavirus referred to other specialties? (never sometimes, regularly, always)

37 Mention which specialties: surgery, gynecology, urology, infectology, dermatology, psychology, psychiatry, otorhinolaryngology, others (never, sometimes, regularly, always)

38 On average, how many follow-up consultations are given to patients with diseases and cancers related to the Human Papillomavirus each year?

39 At your institution, how many patients treated for diseases and cancers related to the Human Papillomavirus have recurrences?

40 Does your institution take photographs of patients with recurrences? (never, sometimes, regularly, always)

41 At your institution, what therapies are considered for the treatment of patients with diseases and cancers related to the Human Papillomavirus when they recur?

42 At your institution, are patients who recur after treatment of diseases and cancers related to the Human Papillomavirus referred to other specialties? (yes, no)

43 To which of the following specialties are patients with recurrences of diseases and cancers related to the Human Papillomavirus referred? Surgery, urology, infectology, dermatology, psychology, psychiatry, otorhinolaryngology, others

Appendix II: Questionnaire for Round II

1- Contact information

2- Of your patients with diseases and cancers related to the Human Papillomavirus, what percentage are: (a) men, (b) women?

Answer:

Men: 70% (50-100)

Women: 30% (0-50)

Do you agree with this answer?

Yes, no.

If you said No, please write the percentage of your patients with HPV-related diseases and cancers who are men and women:

Please justify your answer:

3- Of your patients with diseases and cancers related to the Human Papillomavirus, what percentage is under 20 years old?

Answer:

2% (0-20)

Do you agree with this answer?

Yes, no.

If you said No, please write the percentage of your patients with diseases and cancers related to Human Papillomavirus who are under 20 years of age:

Please justify your answer:

4- Of your patients with diseases and cancers related to the Human Papillomavirus, what percentage presents the condition for the first time?

Answer:

80% (0-50)

Do you agree with this answer?

Yes, no.

If you said No, please write the percentage of your patients with diseases and cancers related to the Human Papillomavirus who present the condition for the first time:

Please justify your answer:

5- Of your patients with diseases and cancers related to the Human Papillomavirus for the first time, what percentage are: (a) men, (b) women?

Answer:

Men: 60% (50-100)

Women: 40% (0-50)

Do you agree with this answer?

Yes, no.

If you said No, please write the percentage of your patients with diseases and cancers related to the Human Papillomavirus who are diagnosed for the first time who are men and women:

Please justify your answer:

6- Of your patients with diseases and cancers related to the Human Papillomavirus for the first time, what percentage is under 20 years old?

Answer: 2% (0-50)

Do you agree with this answer?

Yes, No.

If you said No, please write the percentage of your patients with diseases and cancers related to the Human Papillomavirus for the first time who are under 20 years of age:

Please justify your answer:

7- How many cases of men diagnosed with diseases and cancers related to the Human Papillomavirus are there in Costa Rica annually? Please provide an estimate.

Answer: 95 (40-10010)

Do you agree with this answer?

Yes, No.

If you said No, please provide the estimated number of cases of men diagnosed with diseases and cancers related to the Human Papillomavirus in Costa Rica annually:

Please justify your answer:

8- In the United States, the prevalence of diseases and cancers related to the Human Papillomavirus in men is approximately 11.2 What do you consider to be the prevalence of diseases and cancers related to the Human Papillomavirus in your institution?

Answer: 10 (4-11.2).

Do you agree with this answer?

Yes, No.

If you said No, please provide the prevalence of diseases and cancers related to Human Papillomavirus at your institution:

Please justify your answer:

9- In addition to the clinical review, how often does your institution perform diagnostic tests on patients with diseases and cancers related to the Human Papillomavirus?

Answer:

Never + Sometimes: 60%

Regularly + always: 40%

Please answer the question and choose one of the following options:

Never

Sometimes

Regularly

Always

Please justify your answer:

10- How often are the following diagnostic tests ordered for patients with suspected HPV-related diseases and cancers at your institution?

10.1 HBV Serology

Answer:

Never + Sometimes: 60%

Regularly + always: 40%

Please answer the question and choose one of the following options:

Never

Sometimes

Regularly

Always

Please justify your answer:

10.2 HCV Serology

Answer:

Never + Sometimes: 60%

Regularly + always: 40%

Do you agree with this answer?

Please choose one of the following options:

Never

Sometimes

Regularly

Always

Please justify your answer:

10.3 ELISA for HIV

Answer:

Never + Sometimes: 40%

Regularly + always: 60%

Please choose one of the following options:

Never

Sometimes

Regularly

Always

Please justify your answer:

10.4 VDRL for Syphilis

Answer:

Never + Sometimes: 40%

Regularly + always: 60%

Do you agree with this answer?

Please choose one of the following options:

Never

Sometimes

Regularly

Always

Please justify your answer:

10.5 Penoscopy

Answer:

Never + Sometimes: 60%

Regularly + always: 40%

Please choose one of the following options:

Never

Sometimes

Regularly

Always

Please justify your answer:

10.6 Anoscopy

Answer:

Never + Sometimes: 60%

Regularly + always: 40%

Please choose one of the following options:

Never

Sometimes

Regularly

Always

Please justify your answer:

11- In men with diseases and cancers related to the Human Papillomavirus, what are the diagnostic procedures used in your institution and the average number of visits for each procedure?

11.1 Biopsy

Answer: 2 (0-25)

Do you agree with this answer?

Yes, No.

If you said No, please provide the average number of visits:

Please justify your answer:

11.2 Simple observation

Answer: 3 (0-25)

Do you agree with this answer?

Yes, No.

If you said No, please provide the average number of visits:

Please justify your answer:

11.3 Other procedure

Answers:

Fulguration

Serology

Upper aerodigestive endoscopy

Do you agree with this answer?

Yes, No.

If No, please provide “Other” procedure and average number of visits:

Please justify your answer:

12- Of the previous procedures, which is the most used?

Answer:

60% Biopsy

20% Other

20% Simple observation

Please indicate which is the most used procedure:

Please justify your answer:

13- Of the previous procedures, which is the second most used?

40% Biopsy

40% Simple observation

20% Other

Please indicate which is the second most used procedure:

Please justify your answer:

14- At your institution, are there clinical practice guidelines available for the treatment of diseases and cancers related to the Human Papillomavirus?

Answer:

60% No

40% Yes

Please indicate if at your institution there are clinical practice guidelines available for the treatment of diseases and cancers related to the Human Papillomavirus:

Yes, No.

Please justify your answer:

15- Do you have all the necessary resources for the treatment of diseases and cancers related to the Human Papillomavirus according to the guidelines?

Answer:

60% No

40% Yes

Please indicate if you have all the necessary resources for the treatment of diseases and cancers related to the Human Papillomavirus according to the guidelines:

Yes, No.

Please justify your answer:

16- If the answer to the previous question is “no”, what resources are missing?

Answer:

Screening

Typing

Access to immunotherapy

Biopsy access

Access serological tests

More timely approach

Shorter boarding time

Do you agree with this answer?

Yes, No.

If you said No, please indicate which resources are missing:

Please justify your answer:

17- At your institution, how often do patients with diseases and cancers related to the Human Papillomavirus receive a specific type of treatment?

Answer:

Never + Sometimes: 60%

Regularly + always: 40%

Please choose one of the following options:

Never

Sometimes

Regularly

Always

Please justify your answer:

18- Whether pharmacological or ablative, what treatment is most commonly prescribed as a first line of treatment at your institution? Please rank from the most prescribed (1) to the least (8), please choose a different number for each treatment. Also, provide the average number of visits for each treatment.

18.1: Pharmacological - Trichloroacetic acid.

Results:

Less prescribed: 40%

Most prescribed: 60%

Average visits: 0 (0-3)

Please rate whether this procedure is most prescribed (1) to least prescribed (8):

Please indicate the average number of visits:

18.2 Pharmacological - Podophyllin.

Results:

Less prescribed: 20%

Most prescribed: 40%

Moderately prescribed: 40%

Average visits: 0 (0-3)

Please rate whether this procedure is most prescribed (1) to least prescribed (8):

Please indicate the average number of visits:

18.3 Pharmacological - Imiquimod.

Results:

Less prescribed: 60%

Most prescribed: 20%

Moderately prescribed: 20%

Average visits: 0 (0-3)

Please rate whether this procedure is most prescribed (1) to least prescribed (8):

Please indicate the average number of visits:

18.4 Ablative - Cryotherapy.

Results:

Less prescribed: 20%

Most prescribed: 40%

Moderately prescribed: 40%

Average visits: 0 (0-4)

Please rate whether this procedure is most prescribed (1) to least prescribed (8):

Please indicate the average number of visits:

18.5 Ablative - Excision.

Results:

Less prescribed: 20%

Most prescribed: 60%

Moderately prescribed: 20%

Average visits: 1 (0-4)

Please rate whether this procedure is most prescribed (1) to least prescribed (8):

Please indicate the average number of visits:

18.6 Ablative - Laser surgery.

Results:

Less prescribed: 60%

Moderately prescribed: 40%

Average visits: 0 (0-3)

Please rate whether this procedure is most prescribed (1) to least prescribed (8):

Please indicate the average number of visits:

18.7 Ablative - Others.

Results:

Less prescribed: 20%

Most prescribed: 60%

Moderately prescribed: 20%

Average visits: 0 (0-3)

Please rate whether this procedure is most prescribed (1) to least prescribed (8):

Please indicate the average number of visits:

19- Based on the answers provided above regarding the choice of pharmacological and/or alblative treatments, for what reason(s) is this choice made at your institution?

The three main reasons:

Site and morphology: 100%

Size: 80%

Convenience: 40%

Treatment cost: 40%

Number: 20%

Adverse effects: 20%

Please provide the third most important reason:

Justify your answer:

20- Of the three main reasons chosen above, which reason is the most important?

Answers:

Size: 40%

Site and morphology: 20%

Adverse effects: 20%

Cost: 20%

Please choose the most important reason:

Please justify your answer:

21- Of the three main reasons chosen above, which reason is the least important?

Answers:

40% Site and morphology: 40%

Number: 20%

Convenience: 20%

Cost: 20%

Please choose the least important reason:

Please justify your answer:

22- Of the three main reasons chosen above, which reason is in second place in importance?

Answers:

Size: 40%

Site and morphology: 20%

Convenience: 20%

Place: 20%

Please choose the second most important reason:

Please justify your answer:

23- In your institution, what type of therapy is considered the second line of treatment for diseases and cancers related to the Human Papillomavirus?

Answer:

Chemotherapy: 60%

Radiotherapy: 40%

Vaccination: 20%

Ablation: 20%

Systemic: 20%

Please choose the therapy that is considered the second line of treatment for diseases and cancers related to Human Papillomavirus:

Please justify your answer:

24- How many days on average do patients with diseases and cancers related to the Human Papillomavirus stay in the hospital? (days)

Answer: 7 (0-7)

Do you agree with this answer?

Yes, No.

If you do not agree, please provide the average number of days that patients with diseases and cancers related to the Human Papillomavirus remain in the hospital:

Please justify your answer:

25- Do patients treated for diseases and cancers related to the Papillomavirus have the following side effects?

25.1 Burning

Answer:

Never + Sometimes: 40%

Regularly + always: 60%

Please choose one of the following options:

Never

Sometimes

Regularly

Always

Please justify your answer:

25.2 Itching

Answer:

Never + Sometimes: 40%

Regularly + always: 60%

Please choose one of the following options:

Never

Sometimes

Regularly

Always

Please justify your answer:

25.3 Scab formation

Answer:

Never + Sometimes: 60%

Regularly + always: 40%

Please choose one of the following options:

Never

Sometimes

Regularly

Always

Please justify your answer:

25.4 Discoloration

Answer:

Never + Sometimes: 60%

Regularly + always: 40%

Please choose one of the following options:

Never

Sometimes

Regularly

Always

Please justify your answer:

25.5 Others

Answer:

Never + Sometimes: 40%

Regularly + always: 60%

Please choose one of the following options:

Never

Sometimes

Regularly

Always

Please justify your answer:

26- Do patients treated for diseases and cancers related to the Human Papillomavirus have complications?

Answer:

Never + Sometimes: 60%

Regularly + always: 40%

Please choose one of the following options:

Never

Sometimes

Regularly

Always

Please justify your answer:

27- What is the total annual cost of a patient with a disease and cancer related to the Human Papillomavirus at your institution? (answer in colones). Please provide an estimate.

Answer: 15,918,321 (300 - 20,000,000) colones

Do you agree with this answer?

Yes, No.

If you do not agree, please provide the estimated cost (in colones):

28- What is the annual cost of a patient with a disease and cancer related to the Human Papillomavirus who attends an outpatient clinic at your institution? (answer in colones). Please provide an estimate.

Answer: 195,000 (30 - 10,000,000) colones

Do you agree with this answer?

Yes, No.

If you do not agree, please provide the estimated cost (in colones):

29- What is the annual cost of the treatments received by a patient with a disease and cancer related to the Human Papillomavirus at your institution? (answer in colones). Please provide an estimate.

Answer: 2,000,000 (0 - 5,000,000) colones

Do you agree with this answer?

Yes, No.

If you do not agree, please provide the estimated cost (in colones):

30- How often do patients treated for diseases and cancers related to the Human Papillomavirus have the following complications?

30.1 Deformity

Answer:

Never + Sometimes: 40%

Regularly + Always: 60%

Please choose one of the following options:

Never

Sometimes

Regularly

Always

Please justify your answer:

31- Mention to which specialties you refer patients with diseases and cancers related to the Human Papillomavirus

31.1 Surgery

Answer:

Never + Sometimes: 40%

Regularly + Always: 60%

Please choose one of the following options:

Never

Sometimes

Regularly

Always

Please justify your answer:

31.2 Gynecology

Answer:

Never + Sometimes: 60%

Regularly + Always: 40%

Please choose one of the following options:

Never

Sometimes

Regularly

Always

Please justify your answer:

31.3 Urology

Answer:

Never + Sometimes: 60%

Regularly + Always: 40%

Please choose one of the following options:

Never

Sometimes

Regularly

Always

Please justify your answer:

31.4 Dermatology

Answer:

Never + Sometimes: 60%

Regularly + Always: 40%

Please choose one of the following options:

Never

Sometimes

Regularly

Always

Please justify your answer:

31.5 Psychology

Answer:

Never + Sometimes: 60%

Regularly + Always: 40%

Please choose one of the following options:

Never

Sometimes

Regularly

Always

Please justify your answer:

31.6 Otorhinolaryngology

Answer:

Never + Sometimes: 60%

Regularly + Always: 40%

Please choose one of the following options:

Never

Sometimes

Regularly

Always

Please justify your answer:

31.7 Others

Answer:

Never + Sometimes: 60%

Regularly + Always: 40%

Please choose one of the following options:

Never

Sometimes

Regularly

Always

Please justify your answer:

32- On average, how many follow-up consultations are given to patients with diseases and cancers related to the Human Papillomavirus each year?

Answer: 4 (3-50)

Do you agree with this answer?

Yes, No.

If you do not agree, please provide the number of follow-up consultations given to patients with diseases and cancers related to the Human Papillomavirus each year:

Please justify your answer:

33- At your institution, how many patients treated for diseases and cancers related to the Human Papillomavirus have recurrences?

Answer: 15 (4-50)

Do you agree with this answer?

Yes, No.

If you do not agree, please provide the number of patients treated for diseases and cancers related to Human Papillomavirus who have recurrences:

Please justify your answer:

34- Do you take photographs of patients with recurrences at your institution?

Answer:

Never + Sometimes: 40%

Regularly + Always: 60%

Please choose one of the following options:

Never

Sometimes

Regularly

Always

Please justify your answer:

35- At your institution, what therapies are considered for the treatment of patients with diseases and cancers related to the Human Papillomavirus when they recur?

Surgery: 80%

Radiotherapy: 60%

Electrofulguration: 20%

Chemotherapy: 20%

Local ablation: 20%

Systemic treatment: 20%

Please indicate which therapies are considered for the treatment of patients with diseases and cancers related to the Human Papillomavirus when they recur:

Please justify your answer:

36- To which of the following specialties are patients with recurrences of diseases and cancers related to the Human Papillomavirus referred?

Answers:

Otorhinolaryngology: 80%

Psychology: 60%

Surgery: 40%

Dermatology: 40%

Others: 40%

Urology: 20%

Infectology: 20%

Psychiatry: 20%

Please indicate which specialties patients with recurrences of diseases and cancers related to the Human Papillomavirus are referred to:

# Please justify your answer:
